# Antimicrobial Efficiency of Some Essential Oils in Antibiotic-Resistant *Pseudomonas aeruginosa* Isolates

**DOI:** 10.3390/plants11152003

**Published:** 2022-07-31

**Authors:** Luc Tran Van, Ilinca Hagiu, Adelina Popovici, Florica Marinescu, Irina Gheorghe, Carmen Curutiu, Lia Mara Ditu, Alina-Maria Holban, Tatiana Eugenia Sesan, Veronica Lazar

**Affiliations:** 1 Microbiology & Immunology Department, Faculty of Biology, University of Bucharest, 77206 Bucharest, Romania; luctv@vxut.edu.vn (L.T.V.); a.popovici20@s.bio.unibuc.ro (A.P.); florica.marinescu@bio.unibuc.ro (F.M.); irina.gheorghe@bio.unibuc.ro (I.G.); lia-mara.ditu@bio.unibuc.ro (L.M.D.); alina.m.holban@bio.unibuc.ro (A.-M.H.); tatianasesan@yahoo.com (T.E.S.); veronica.lazar@bio.unibuc.ro (V.L.); 2 Research Institute of the University of Bucharest-ICUB, University of Bucharest, 050657 Bucharest, Romania; 3 The Overlake Private School, 108th St., Redmond, WA 98053, USA; ilincah@hotmail.com

**Keywords:** *Pseudomonas aeruginosa*, virulence markers, antibiotic-resistant strains, essential oils, alternative antimicrobials, tea tree, thyme, sage, eucalyptus

## Abstract

*Pseudomonas aeruginosa* is a non-fermentative Gram-negative opportunistic pathogen, frequently encountered in difficult-to-treat hospital-acquired infections and also wastewaters. The natural resistance of this pathogen, together with the frequent occurrence of multidrug-resistant strains, make current antibiotic therapy inefficient in treating *P. aeruginosa* infections. Antibiotic therapy creates a huge pressure to select resistant strains in clinical settings but also in the environment, since high amounts of antibiotics are released in waters and soil. Essential oils (EOs) and plant-derived compounds are efficient, ecologic, and sustainable alternatives in the management of various diseases, including infections. In this study, we evaluated the antibacterial effects of four commercial essential oils, namely, tea tree, thyme, sage, and eucalyptus, on 36 *P. aeruginosa* strains isolated from hospital infections and wastewaters. Bacterial strains were characterized in terms of virulence and antimicrobial resistance. The results show that most strains expressed soluble pore toxin virulence factors such as lecithinase (89–100%) and lipase (72–86%). All *P. aeruginosa* strains were positive for alginate encoding gene and 94.44% for protease IV; most of the strains were exotoxin producers (i.e., 80.56% for the ExoS gene, 77.78% for the ExoT gene, while the ExoU gene was present in 38.98% of the strains). Phospholipase-encoding genes (plc) were identified in 91.67/86.11% of the cases (plcH/plcN genes). A high antibiotic resistance level was identified, most of the strains being resistant to cabapenems and cephalosporins. Cabapenem resistance was higher in hospital and hospital wastewater strains (55.56–100%) as compared to those in urban wastewater. The most frequently encountered encoding genes were for extended spectrum β-lactamases (ESBLs), namely, *bla*_CTX-M_ (83.33% of the strains), *bla*_SHV_ (80.56%), *bla*_GES_ (52.78%), and *bla*_VEB_ (13.89%), followed by carbapenemase-encoding genes (*bla*_VIM_, 8.33%). Statistical comparison of the EOs’ antimicrobial results showed that thyme gave the lowest minimum inhibitory concentrations (MIC) and minimum biofilm eradication concentrations (MBEC) in *P. aeruginosa*-resistant isolates, making this EO a competitive candidate for the development of efficient and ecologic antimicrobial alternatives.

## 1. Introduction

*Pseudomonas aeruginosa* is a versatile Gram-negative, aerobic, rod-shaped bacterium that belongs to the bacterial family Pseudomonadaceae, a member of γ-proteobacteria [1]. A recognized opportunistic pathogen, *P. aeruginosa* is responsible for severe hospital-acquired (nosocomial) and community infections, being present in chronic lungs of cystic fibrosis patients. The bacterium’s virulence depends on many cell-associated and extracellular factors, which enable host colonization, survival, invasion, and persistent infections [2]. Attached virulence factors, such as lipopolysaccharide, pili, and flagella are involved in tissue colonization, while soluble virulence factors such as alkaline protease, elastase, exotoxin A, pyoverdine, pyocyanin, and rhamnolipids are responsible for host immune evasion and persistence [3]. The ability to develop biofilms in various environments, including chronic lung infections and on implantable devices, makes *P. aeruginosa* the preferred Gram-negative model to investigate biofilm regulation. The study of bacterial biofilms is a medical priority since these multicellular-attached microbial communities are highly tolerant to increased antibiotic concentrations and host immune defense [2,3].

It has been estimated that at least 70% of the persistent *P. aeruginosa* infections are biofilm associated. Microbial tolerance associated with biofilm growth, its natural resistance mechanisms, and the acquisition of multiple resistance genes have led this opportunistic pathogen to be categorized as a superbug [2].

Multiple antibiotics administered in high amounts are often required to treat *P. aeruginosa* infections in critical patients. The administration of high amounts of antibiotics in clinical settings, together with their use in some agriculture and animal facilities, puts huge pressure on the selection of antibiotic-resistant strains [4]. Large amounts of antibiotics are released into hospital and municipal wastewaters due to incomplete metabolism in humans and animals or due to inappropriate disposal of unused drugs [5]. These substances finally find their ways into different natural environmental compartments. 

Antibiotic-resistant genes, antibiotic-resistant bacteria, and antibiotic residues have been detected in wastewaters from community, hospitals, and animal slaughterhouses [6,7,8,9]. These factors create a high selective pressure for the development of multi-resistant bacteria strains and support the increased resistance rates [10].

In the context of high antibiotic resistance rates, alternatives to handling difficult-to-treat *P. aeruginosa* infections, caused by resistant strains, are urgently required. In recent years, researchers have investigated alternative physical, chemical, or biological alternatives to fight resistant bacteria, such as: (i) virulence modulators (including biofilm formation inhibitors), (ii) antimicrobial vaccines, (iii) genetically engineered bacteria, (iv) bacteriophages, (v) plant-derived compounds and essential oils (EOs), (vi) nanoparticles [11,12], (vii) plasmas, and (viii) other physic microorganism removal methods based on light or ultrasound [13].

Virulence modulation represents an essential approach in dealing with antimicrobial-resistant strains. Targeting specific virulence features would enable the control of the infectious process, and this approach could be efficient in both susceptible and antibiotic-resistant strains. Furthermore, natural plant-derived compounds and mixtures, i.e., EOs, were intensively investigated for the development of efficient antimicrobials to overcome high antibiotic resistance rates in *P. aeruginosa* [14,15].

Several natural and synthetic compounds have been introduced in previous studies to modulate *P. aeruginosa* growth and virulence. Extracts of various natural products and EOs of certain plants (i.e., lavender, eucalyptus, and citrus) have shown inhibitory effects against the growth and virulence of *P. aeruginosa* [16,17]. EOs are volatile compounds produced by the secondary metabolism of plants, mainly composed of terpenoids, terpenes, and aliphatic and aromatic constituents, and they have been proven to be efficient antimicrobial agents in multi-resistant bacteria and fungi [18,19] due to a broad spectrum of biocidal activity [20,21,22]. EO composition, such as phenolic content and antioxidants, significantly influence their antimicrobial properties [23]. 

Sage (*Salvia officinalis*), thyme (*Thymus vulgaris*), and *Eucalyptus* sp. are among the most important medicinal plants, used from ancient time as aromatic plants, but also in traditional medicine. Recent studies have shown that the essential oils produced by these plants, along with tea tree oil extracted from *Melaleuca alternifolia*, have antibacterial and antifungal effects against various pathogenic microorganisms. The total content of EOs as well as their chemical composition can vary depending on environmental factors, growth region, phenological cycle, and cultivation practices. Alpha- and beta-thujone; 1,8-cineole; camphor; and humulene are among most identified compounds in *Salvia officinalis* EO. Thyme essential oils (TEOs) include thymol, carvacrol, and paracymene. The antibacterial activity of *Eucalyptus* extracts is due to components such as 1,8-cineole, citronellal, citronellol, citronellyl acetate, p-cymene, eucamalol, limonene, linalool, β-pinene, γ-terpinene, α-terpinol, alloocimene, and aromadendrene. Tea tree oil consists of about 100 terpinenes and their alcohols, of which terpinene-4-ol, α-pinene, linalool, and α-terpineol are considered the most important for antimicrobial activity [24,25,26,27,28].

In this study, we aimed to evaluate the antimicrobial activity of four EOs with high content of antibacterial compounds on multidrug-resistant *P. aeruginosa* strains. Commercial tea tree, thyme, sage, and eucalyptus EOs were evaluated on planktonic and biofilm *P. aeruginosa* cultures obtained from hospital-acquired infections and wastewaters. Correlations between microbial isolation sources, virulence and resistance profiles, and antimicrobial efficiency of the tested EOs were also established.

## 2. Results

### 2.1. The Characterization of P. aeruginosa Strains

This study included 36 strains; two laboratory strains, namely, *Pseudomonas aeruginosa ATCC 27853* and *PA01*, were purchased from American Type Culture Collection (ATCC, US), while 34 strains were isolated from clinical infections (*n* = 7), hospital wastewater (*n* = 9), and municipal wastewater (*n* = 18) from Romania.

Nine strains were isolated from untreated or treated (chlorine treatment) hospital wastewater from two large hospital units from Bucharest, while 18 strains were isolated from the urban wastewaters of large Romanian cities from the northeast of the country.

#### 2.1.1. Antibiotic Susceptibility

**Figure 1:**
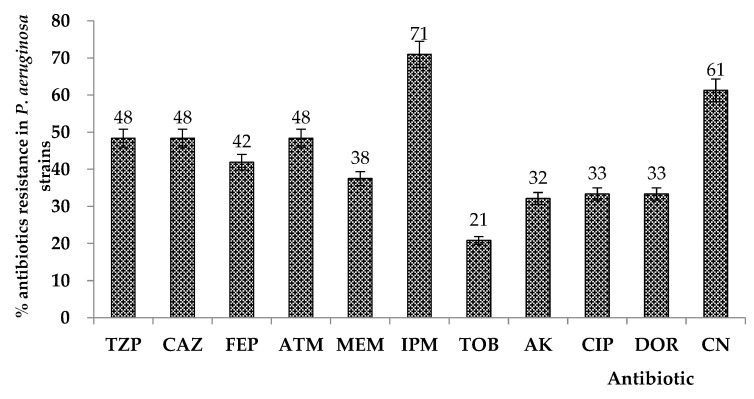
Results of antibiotic resistance of *Pseudomonas aeruginosa*-evaluated strains (*n* = 36). (TZP: piperacillin/tazobactam; CAZ: ceftazidime; FEP: cefepime; ATM: aztreonam; MEM: meropenem; IPM: imipenem; TOB: tobramycin; AK: amikacin; CIP: ciprofloxacin; DOR: doripenem; CN: gentamicin).

Antibiotic susceptibility was evaluated by the disc-diffusion method. The results showed high levels of antibiotic resistance in the analyzed *P. aeruginosa* strains. Antibiotic resistance to piperacillin/tazobactam (TZP) was encountered in 48% of the analyzed strains, ceftazidime (CAZ) in 48%, cefepime (FEP) in 42%, aztreonam (ATM) resistance was observed for 48% of the *P. aeruginosa* strains, meropenem (MEM) in 38%, imipenem (IPM) in 71%, tobramycin (TOB) in 21%, amikacin (AK) in 32%, ciprofloxacin (CIP) in 33%, doripenem (DOR) in 33%, and Gentamicin (CN) in 48%, as shown in Figure 1.

The results demonstrated that the analyzed *P. aeruginosa* strains possess increased resistance to imipenem, but also gentamicin, aztreonam, ceftazidime, and the piperacillin/tazobactam combination.

**Figure 2:**
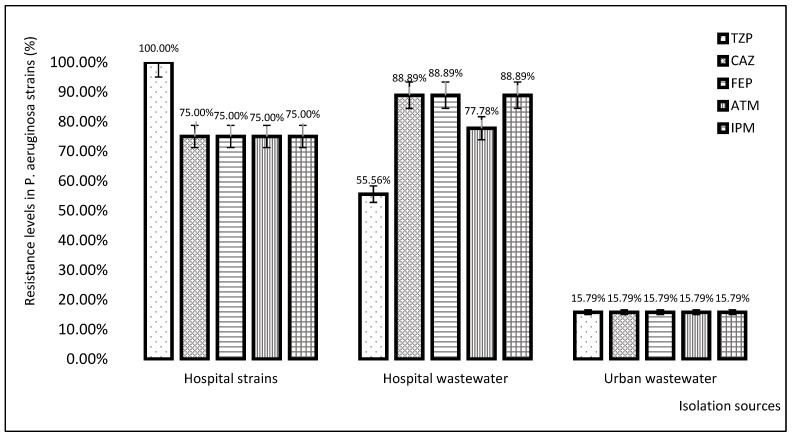
The antibiotic resistance levels (%) of *P. aeruginosa* strains depending on their isolation sources.

Analysis of antibiotic resistance results of strains isolated from various sources (Figure 2) showed that *P. aeruginosa* strains isolated from nosocomial infections and hospital wastewaters account for higher resistance levels (55.56–100%) as compared with urban wastewaters (15.79%).

**Figure 3:**
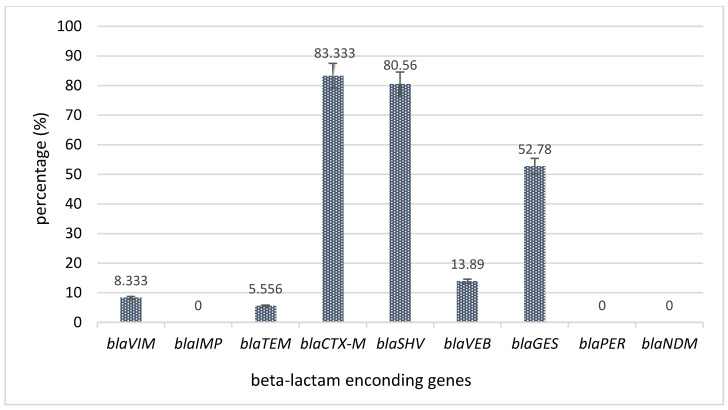
The carbapanamase- and ESBL-encoding genes (%) expressed by *P. aeruginosa* strains.

Indeed, the molecular investigation of the genes encoding for antibiotic resistance revealed that the most frequently encountered encoding genes were for extended spectrum β-lactamases (ESBLs), namely, *bla*_CTX-M_ (*n* = 30 strains), *bla*_SHV_ (*n* = 29 strains), *bla*_GES_ (*n* = 19 strains), and *bla*_VEB_ (*n* = 5 strains), followed by carbapenemase-encoding genes (*bla*_VIM_, *n* = 3 strains) (Figure 3).

#### 2.1.2. Soluble Enzymatic Virulence Factors

**Figure 4:**
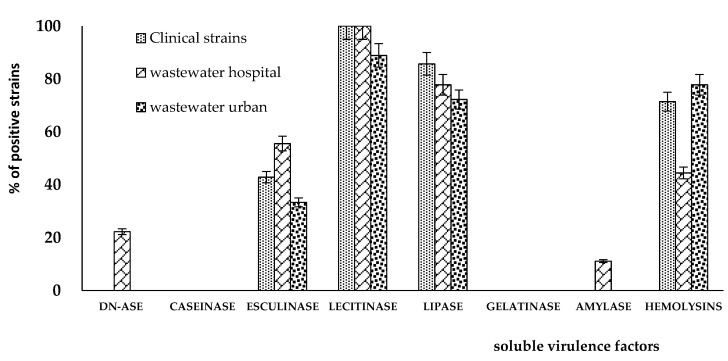
Graphic representation of the distribution of soluble virulence factors expressed by the *P. aeruginosa* strains. The graph represents the percentage (%) of positive strains for each of the evaluated virulence factor depending on their isolation source.

Regarding their virulence profile, the evaluation of soluble virulence factors showed that most of the *P. aeruginosa* strains expressed pore forming enzymes, such as hemolysins, lipase, and lecithinase. The distribution of virulence factors seems to be unrelated to the isolation source of analyzed *P. aeruginosa* strains (Figure 3). In addition, about 50% of the analyzed strains showed the ability to degrade esculin in vitro (Figure 3), and this phenotype could be related to the ability of these strains to acquire iron and produce siderophores [29]. Although not significant, some modifications in the distribution of soluble virulence factors were observed among *P. aeruginosa* strains, depending on their isolation source. Generally, clinical and hospital wastewater isolates showed a higher percentage of positive strains as compared to urban wastewater isolates (Figure 4). Differences in virulence characteristics of microbial strains isolated from various sources were also reported in earlier studies, and they may be explained by strain diversity during infection in particular hosts [30,31]. 

**Figure 5:**
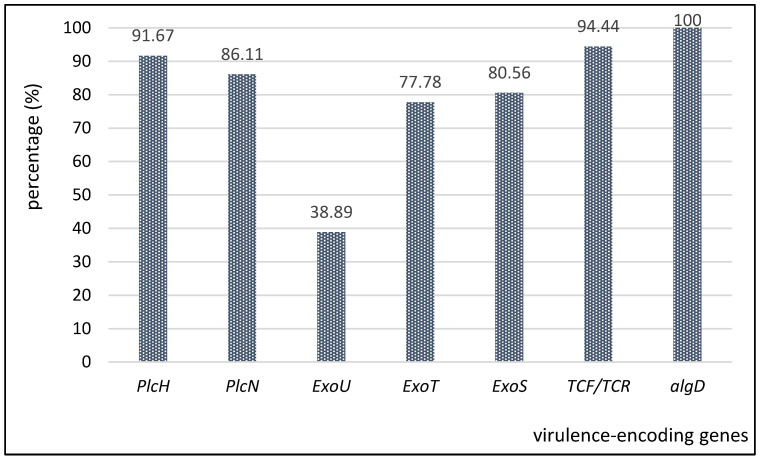
Graphic representation of the distribution of virulence-encoding genes expressed by the *P. aeruginosa* strains.

Regarding the distribution of virulence markers in clinical and wastewater *P. aeruginosa* strains, the molecular analysis showed that 100% of the strains were positive for alginate-encoding gene and 94.44% for *protease IV* (TCF/TCR); the greatest part of the investigated strains were exotoxin producers (i.e., 80.56% for *exoS* gene, 77.78% for *exoT* gene, while the *exoU* gene was present only in 38.98% of the strains). Phospholipase-encoding genes (*plc*) were revealed as follows: *plcH* in 91.67% and *plcN* in 86.11% of the analyzed strains (Figure 5).

### 2.2. Antimicrobial Potential of Essential Oils 

The next step of this research was to analyze the antimicrobial potential of four essential oils (tea tree, thyme, sage, and eucalyptus) by quantitative protocols to evaluate their antimicrobial potential to be considered as alternative and ecologic antimicrobials [32]. 

**Figure 6:**
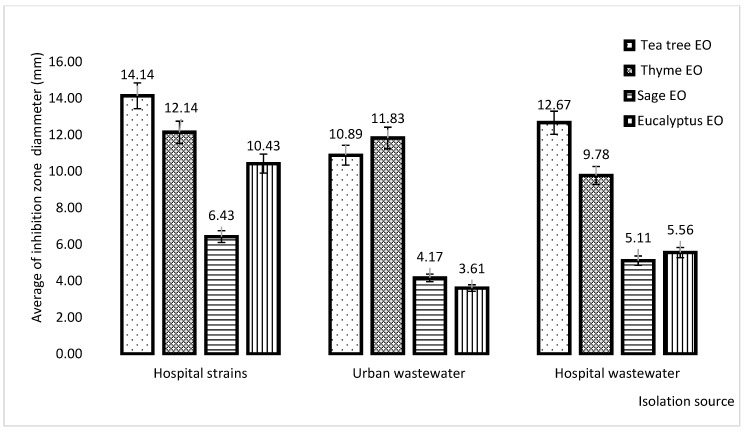
Graphic representation of the average inhibition diameter zone of the EOs in *P. aeruginosa* strains, regardless their isolation source.

The qualitative analysis revealed that the large diameter of the inhibition zone was observed in cases of tree tea EO, followed by thyme EO regardless of isolation sources (Figure 6). 

Quantitative assays revealed two key parameters, namely, minimum inhibitory concentration (MIC), and minimum biofilm eradication concentration (MBEC). These were utilized to show the efficiency of the analyzed EOs in planktonic (free floating) and attached (biofilms) bacteria cultures. 

**Figure 7:**
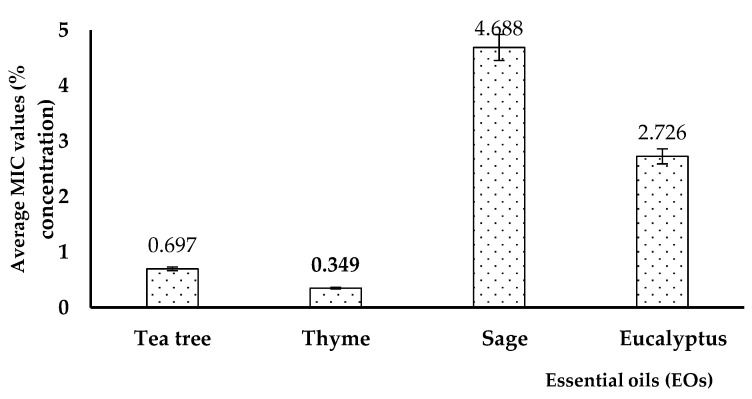
Average minimum inhibitory concentration (MIC) values, expressed as percent (%) concentration of the EOs in *P. aeruginosa* strains, regardless of their isolation source.

The results gave various MIC values, depending on the analyzed EO, being influenced also by the isolation source of the *P. aeruginosa* strains. Generally, the lowest MIC values were obtained for thyme and tea tree EOs in the tested *P. aeruginosa* strains, regardless of their isolation source. The average value of the minimum concentration of tea tree was 0.697%; for thyme it was 0.349%, for sage it was 4.688%, while for eucalyptus, it was 2.726%, as shown in Figure 7. 

**Figure 8:**
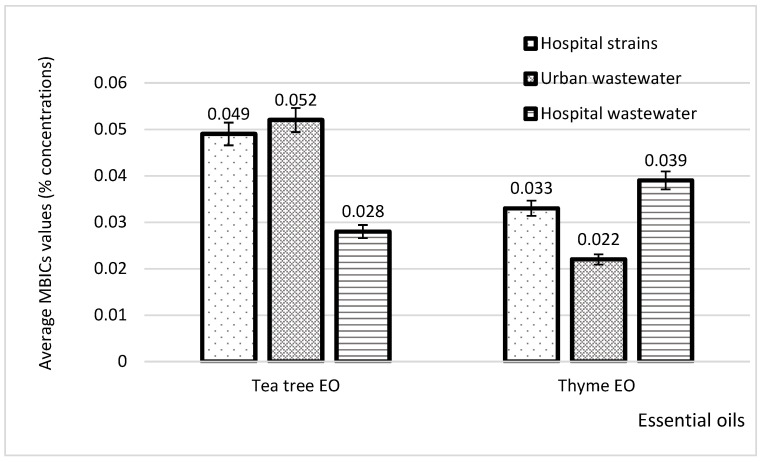
The average MIC values of *P. aeruginosa* strains depending on their isolation source (MICs are expressed as percentage (%) oil in the culturing media).

Next, we compared the minimum inhibitory concentrations of *P. aeruginosa* strains depending on the isolation source of the strains. The results in Figure 8 show that the mean value of the MICs for tea tree EO ranged from 0.538 to 0.759%; for thyme it was 0.246–0.470%, for sage it was 4.583–5.000%, and for eucalyptus it was 2.361–3.214%. Generally, the lowest MICs were found for thyme, followed by tea tree EOs. This trend was constant among all *P. aeruginosa* isolates, regardless of their isolation source. 

**Figure 9:**
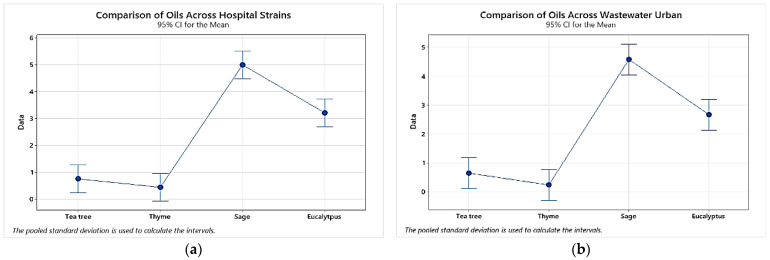

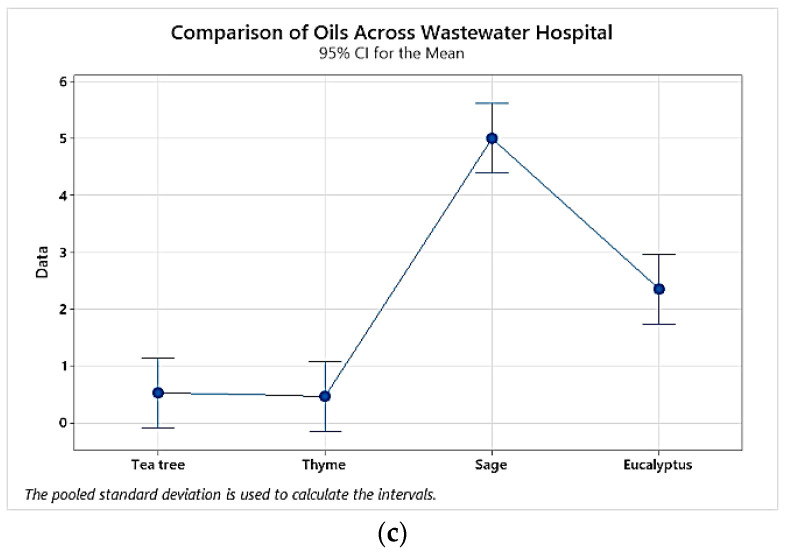
Charts showing comparison of the MIC values for the tested EOs among *P. aeruginosa* strains isolated from different sources: (**a**) comparison of EO MICs across hospital strains, (**b**) comparison of EO MICs across wastewater hospital, (**c**) comparison of EO MICs across wastewater urban; *p* < 0.05 when comparing average MICs of thyme or/and tea tree EOs to sage or/and eucalyptus.

Moreover, we used the Minitab software to statistically reveal the differences among the obtained results. Figure 9 reveals charts showing Minitab comparison of the *p*-values and t-values of the MICs of essential oils in the analyzed *P. aeruginosa* groups, in which a 95% confidence interval (CI) was assumed.

Our data demonstrated the enhanced antibacterial activity of the thyme EO in *P. aeruginosa* multidrug-resistant strains, as previously demonstrated [33]. It seems that MIC values of thyme EO were very low in all the analyzed *P. aeruginosa* groups, regardless of the isolation source. The average MICs obtained for thyme and tea tree EOs were significantly lower as compared to the average MICs of sage and eucalyptus in the analyzed *P. aeruginosa* strains (Figure 4). 

**Figure 10:**
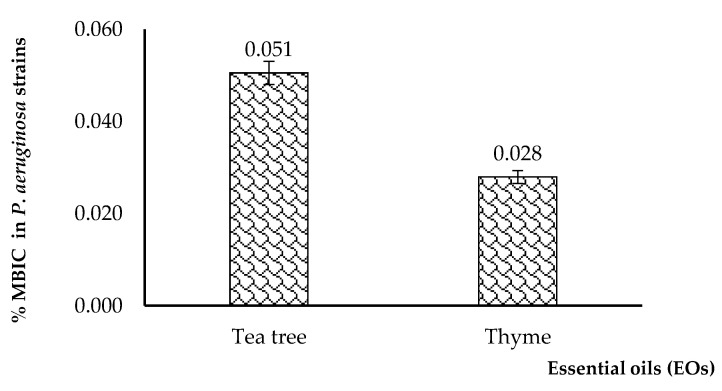
Comparison of the average MBICs of tea tree and thyme EOs (%) in all the analyzed *P. aeruginosa* strains. *p* < 0.05 when comparing average MBICs of thyme to tea tree EOs.

Since the highest antibacterial efficiency in planktonic cultures, expressed as MIC value, was given by tea tree and thyme EOs, we utilized these two EOs for highlighting their impact in *P. aeruginosa* biofilms. It is well known that biofilm formation is a virulence and persistence trait of this opportunistic pathogen [34,35]. The minimum biofilm inhibition concentration (MBIC) assay revealed that thyme is more efficient than tea tree EO, the first being able to inhibit monospecific biofilms at lower concentrations. Our results demonstrated that the average MBIC value obtained for thyme EO in the analyzed *P. aeruginosa* strains was 0.028%, while tea tree EO gave an average MBEC of 0.051%, as shown in Figure 10.

**Figure 11:**
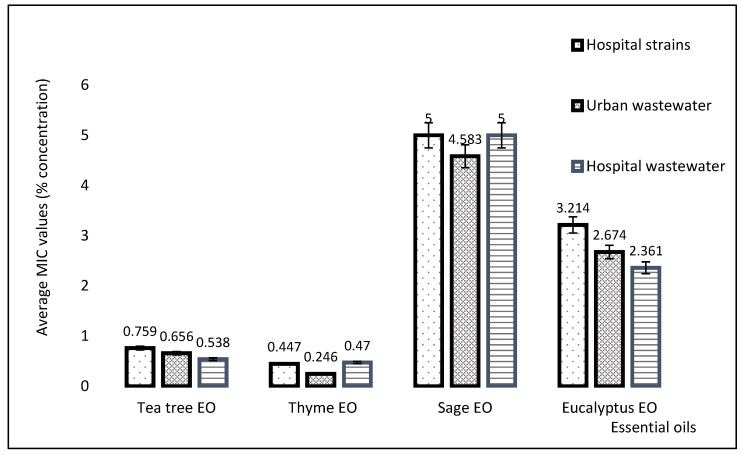
Comparison of the MBICs of thyme and tea tree EOs in *P. aeruginosa* strains depending on their isolation source.

When comparing the MBIC values of the two tested essential oils with the origin of *P. aeruginosa* strains, the results showed that the average value of tea tree was in the range of 0.028–0.052%, and that of thyme was in the range of 0.022–0.039%, as Figure 11 reveals. 

**Figure 12:**
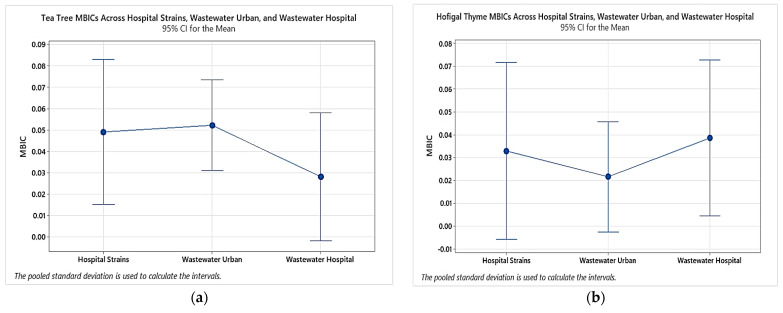
The chart of MBEC values of essential oils from different sources: (**a**) comparison of MBEC values of tea tree from different sources, (**b**) comparison of MBEC values of thyme from different sources.

We found no statistically significant correlation regarding the MBICs of the tested EOs (thyme and tea tree) among *P. aeruginosa* strains isolated from different sources (Figure 12).

**Figure 13:**
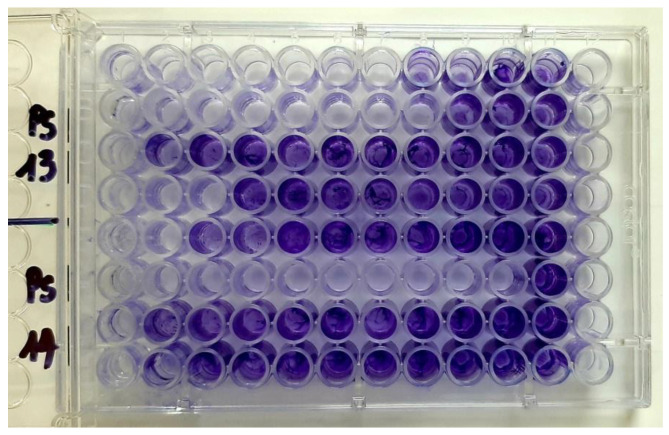
Aspect of 96 well plates after CV staining in cases of 2 *P. aeruginosa* strains treated with EOs (last column (12)—sterility control, the row before (11)—growth control).

However, overall biofilm inhibition efficiency was higher for thyme EOs, since the average MBIC for this oil was significantly lower than the average MBIC obtained for tea tree in the evaluated *P. aeruginosa* strains (Figure 13). 

These data suggest that the analyzed EOs could be highly efficient in both planktonic and biofilm cultures, and also in antibiotic-resistant strains, which are frequent in clinical settings, but also in the environment and wastewaters. 

## 3. Discussion

*P. aeruginosa* is one of the most versatile and naturally resistant opportunistic pathogens. This Gram-negative bacteria model is also recognized for its ability to develop biofilms and a high tolerance to antimicrobials and host immune systems [36]. Our study showed that the strains isolated from Romanian wastewaters and also hospitals presented high resistance rates to most of the tested antibiotics. These findings are in accordance with previous studies reported by our group [37,38,39]. Our results showed a slight reduction in the susceptibility levels of *P. aeruginosa* to cabapenem antibiotics (imipenem, meropenem, doripenem), of which the imipenem resistance level (IPM) is worth mentioning (71%), as compared to previously reports [18,39]. 

Very recently, Buzila et al. demonstrated in a retrospective 11-year study from North-Eastern Romania a very similar imipenem resistance level in *P. aeruginosa* strains recovered from various intra-hospital infections [40]. 

Similar to our obtained results, Tatu et al., in 2017, showed comparable resistance levels to third- and fourth-generation cephalosporins (ceftazidime and cefepime) in *P. aeruginosa* strains recovered from ICU patients admitted in one large hospital unit in Bucharest [41]. Saviuc et al., in 2016, highlighted a comparable resistance level in MDR *P. aeruginosa* nosocomial strains isolated from the same hospital unit (75%) and demonstrated the presence of the *bla*_TEM_ ESBL-encoding gene in analyzed strains [38]. Merezeanu et al. and Porumbel et al., in 2016, demonstrated a high repertoire of virulence markers (i.e., plcH, exoU, exoT, exoS, algD) and a lower resistance level to carbapenems encoded by the *bla*_IMP_ gene and a significant collection of *P. aeruginosa* recovered from hospitalized patients in Bucharest [42,43]. In 2015, Dortret et al. revealed also a high resistance level to carbapenem in *P. aeruginosa* strains isolated from intra-hospital infections from the central part of our country (Cluj Napoca Infectious Disease hospital), demonstrating the spread of carbapenem-resistant clones in Romania and their corresponding carbapenemase encoding genes: ST 2026-VIM-2-producing *P. aeruginosa*, a very related clone to the epidemic ST233 clone; and the ST 1982-IMP-13-producing strain, associated with class 1 integrons [44]. Other authors also reported during that period a comparable carbapenem resistance level encoding by different carbapenemase-encoding genes (i.e., VIM-4; VIM-2) in *P. aeruginosa* strains isolated from hospitalized patients in the south or northeast of Romania [45,46]. This result is somewhat different than the conclusion from the study of Khilasa Pokharel et al. (2019) showing that antibiotics such as imipenem, meropenem, piperacillin/tazobactam, ciprofloxacin, gentamicin, amikacin, and tobramycin are considered to be good choices for treating infections caused by this organism [47]. 

Production of virulence traits is a survival approach for pathogens to evade the host immune mechanism, resulting in pathogenesis mostly at the initial stage of colonization and acute infection. A large amount of virulence factors as well as cell-associated or secreted compounds of minimal and high molecular weight have been documented as vital in establishing infections by *P. aeruginosa* [48]. Along with high acquired resistance rates, *P. aeruginosa* also presents virulence factors that are responsible for their ability to colonize, persist, and invade the host. Biofilm formation represents the most important virulence trait [49], enabling chronic and persistent infections that are highly tolerant to antimicrobials and host defenses [50]. Additionally, enzymatic virulence factors are essential for the progress of infectious processes. Our results showed that almost all *P. aeruginosa* tested strains isolated from all sources produced lipases and lecithinases, which are pore-forming toxins [51]. Correlating virulence profiles and infection clinical outcomes could be very useful for setting up efficient preventive and therapeutic procedures for patients with positive *P. aeruginosa* cultures [51]. 

The pathogenicity *of P. aeruginosa* is multifactorial; the detection of different virulence genes in *P. aeruginosa* isolates suggests that they can be linked with different levels of inherent virulence and their propensity to cause infection. However, it is worth noting that virulence and other phenotypic traits such as resistance genes can contribute to the survival of organisms, as well as in disease spread and severity.

In the context of increasing antibiotic resistance rates, alternative antimicrobial strategies are urgently needed [52]. One efficient approach is to utilize agents to modulate bacterial virulence (i.e., production of virulence enzymes, toxins, and biofilm formation ability) without interfering with microbial fitness [53]. This approach would contribute to lower the rates of selecting resistant mutants, because most of the virulence factors needed for bacteria in the progression of the infectious process are not vital molecules [54]. Therefore, their modulation would not impact the population fitness, which is one of the major triggers for resistant mutant selection [55,56,57]. In our study we demonstrate that the tested EOs could impact *P. aeruginosa* biofilm formation when utilized in subinhibitory concentrations. This aspect could be further exploited in investigating natural factors to control this important virulence factor and control the infectious process and transition to persistent diseases. The fact that the MBICs of thyme and tea tree EOs are very reduced (ranging from 0.051% to 0.028%) in both susceptible and antibiotic-resistant strains, present in wastewaters and clinical samples, serves to suggest these natural products as efficient alternatives in the management of resistant *P. aeruginosa* infections. Our results concluded that EOs are effective, ecological, and sustainable alternatives, which could be used for the control of various infections, including those involving resistant pathogens.

## 4. Materials and Methods

### 4.1. Microbial Strains and Growth Conditions

*Pseudomonas aeruginosa* ATCC 27853 and PAO1 strains were purchased from American Type Culture Collection (ATCC, Manassas, Virginia, US), and 34 strains of *P. aeruginosa* were isolated from intrahospital infections (HAIs) (*n* = 7), hospital sewage tanks from two big hospital units from Bucharest encoded 1 and 2 (*n* = 9), and municipal (urban) wastewaters (*n* = 18) from Bucharest, Romania, during 2018–2020 (Table 1). All strains used were identified by the Maldi-Tof Mass Spectrometry method, both those from hospital infections and those isolated from wastewater. Strains from hospital sewage tanks and urban wastewater were isolated by the membrane filtration method using chromogenic culture media supplemented with antibiotic (ChromID ESBL agar and ChromID CARBA agar; BioMérieux SA, Marcy-l’Etoile, France) for the selection of antibiotic-resistant *P. aeruginosa* strains.

Bacteria strains are maintained as glycerol stocks in the Laboratory of Microbiology, Faculty of Biology, and University of Bucharest, Romania. Glycerol stocks were streaked on nutritive agar to obtain fresh cultures to be used for all further studies.

### 4.2. Essential Oils (EOs)

The study was performed employing the following four EOs: thyme (*Thymus vulgaris),* eucalyptus (*Eucalyptus globulus Labillardiere*), sage (*Salvia officinalis*), and tea tree (*Tea Melaleuca alternifolia*). The EOs were diluted as 1% stock solutions in dimethylsulfoxide (DMSO). Additionally, DMSO was used as a control for the experiments. The EOs were obtain from a Romanian distributor (Hofigal, https://hofigal.eu/, accessed on 24 July 2022), and all the other chemical used were purchased from Sigma–Aldrich Co., Baden-Württemberg, Germany, through MERCK, Romania. 

### 4.3. Characterization of Microbial Strains

#### 4.3.1. Antibiotic Susceptibility (Disc Diffusion Kirby Bauer Method)

The phenotypic susceptibility assay was performed by the disc-diffusion method, as recommended by CLSI 2020 [58] for *P. aeruginosa*.

#### 4.3.2. Evaluation of Soluble Enzymatic Factors Implicated in Bacterial Virulence

The expression of the soluble virulence factors was highlighted by biochemical tests, using specific culture media, with built-in substrate, following a protocol previously reported by Georgescu et al. (2016) [50] and Preda et al. (2021) [31].

A number of eight soluble virulence factors was analyzed: amylase, lecithinase, lipase, caseinase, gelatinase, DNase, hemolysin, and esculin hydrolysis.

Briefly, bacterial strains were seeded on agar containing the substrate for each of the mentioned enzyme, and the as-prepared Petri dishes were incubated for 24–48 h at 37 °C. After incubation, results were read as a visible manifestation of the substrate (i.e., hemolysis, precipitate, halo, color change) appearing around the inoculated bacteria colonies. 

#### 4.3.3. Molecular Characterization of The Strains 

##### DNA Extraction

The DNA from *P. aeruginosa* strains was extracted using an adapted alkaline extraction method. More specifically, 1–5 colonies of bacterial cultures were suspended in 1.5 mL tubes containing 20 µL solution of NaOH 0.05 M (sodium hydroxide) and SDS 0.25% (sodium dodecyl sulphate) and heated on a thermoblock at 95 °C for 15 min for the permeabilization of the bacterial cell wall. The following step was the addition of 180 μL of TE buffer (TRIS + EDTA) 1X and centrifugation at 13,000 rpm for 3 min. After centrifugation, the supernatant was taken in a new tube and store at −20 °C until use [31,50]. 

##### PCR for Virulence and Antibiotic Resistance Genes Identification

Genomic DNA was used as a template for the PCR screening of 7 virulence genes encoding for protease IV (TCF/TCR), three exoenzymes (exoS, exoT, exoU), two phospholipases (plcH (hemolytic phospholipase C), plcN (non-hemolytic phospholipase C), and for alginate (algD), and also for β-lactam (ESBL and carpapenemase encoding genes), aminoglycosides, and quinolone resistance. Primers and amplification programs are presented in Table 2, Table 3, Table 4 and Table 5 [39,42,43]. The amplification products were visualized by electrophoresis on a 1% agarose gel stained with the SYBRSafe DNA and specific weight marker (100pb, Ladder, ThermoScientific, Bucharest, Romania).

### 4.4. Antimicrobial Evaluation of the EOs

#### 4.4.1. Antimicrobial Qualitative Assessment

The in vitro qualitative screening of the antimicrobial activity of the tested EOs was performed by an adapted agar diffusion technique (as recommended by CLSI, 2020) as previously described [59]. Briefly, we obtained a bacterial suspension of 0.5 McFarland density (1.5 × 10^8^ CFU (colony forming units)/mL) in PBS (phosphate buffered saline) from each *P. aeruginosa* fresh culture developed on nutritive agar. Microbial suspensions were utilized to swab inoculate Mueller Hinton agar distributed in 90 mm diameter Petri dishes. Five microliters of each EO, prepared as a 1% solution in DMSO (dimethyl sulfoxide), was drop-added on the *P. aeruginosa*-inoculated Petri dishes and then incubated for 16–18 h at 37 °C.

The results were explained by measuring the diameters of the inhibitory regions that appear around the EOs and denoting them as "mm".

#### 4.4.2. Minimum Inhibitory Concentration (MIC) Evaluation (Microdilution Method)

For establishing the MIC (minimum inhibitory concentration) values of the evaluated EOs, we utilized a microdilution method performed in nutritive broth. The sterile broth was added in sterile 96 well plates, and binary dilutions of each tested EO were performed in a final volume of 150 μL. After realizing the binary dilutions, 15 μL of microbial suspension adjusted to an optical density of 0.5 McFarland (1.5 × 10^8^ CFU/mL) was added in each well. The MIC values were established by naked eye analysis and spectrophotometric measurement (Absorbance at 600 nm). 

#### 4.4.3. Biofilm Formation and MBEC (Minimum Biofilm Eradication Concentrations) of the EOs

Monospecific biofilm formation was evaluated by the crystal violet microdilution method. Briefly, 96-multi well plastic plates containing binary dilutions of the tested EOs in a final volume of 150 µL in nutritive broth were inoculated with 15 µL microbial suspensions of 108 CFU/mL prepared in sterile saline. The as-prepared plates were incubated for 24 h at 37 °C. After incubation, the wells were discarded, washed with PBS to remove unattached cells, and the bacterial cells adhered to the plastic walls were stained by 1% violet crystal solution for 15 min. The colored biofilm was thereafter fixed by cold methanol for 5 min and resuspended by 33% acetic acid solution. The Abs at 490 nm of the blue suspension was measured using an ELISA reader. The obtained absorbance values were proportional with the intensity of biofilm formation on the wells of the plates. MBICs (minimum biofilm inhibitory concentrations) were established by dividing the average Abs 490 value for untreated control by two. The Abs 490 nm/2 of the untreated control represents the reference value for the calculation of MBEC for each EO. Therefore, MBECs for each of the tested EOs was established as the lowest concentration that gives an Abs 490 nm value corresponding to (less than) half of the Abs 490 nm value of the untreated control.

Each experiment was performed in triplicate and repeated on at least three separate occasions.

### 4.5. Statistical Analysis of the Results

Statistical analysis was performed in Minitab^®^ Statistical Software, with a significance level of *p* < 0.05 (95% confidence level). In order to be able to calculate the significance in two sample-paired t-tests, all MIC and MBIC values that were not quantifiable (>50%) were considered as the next two-fold dilution factor (100%).

## 5. Conclusions

This study reports on virulence and resistance characterization of recently isolated *P. aeruginosa* strains obtained from areas of concern in Romania, such as hospital infections, hospital sewage, and wastewater treatment plants. Although profiling of antibiotic resistance and virulence factors has been done in the past, constant updating is needed for learning about virulence changes and the occurrence of resistant strains in this highly versatile opportunistic pathogen.

Since antibiotic-resistant *P. aeruginosa* represents one of the most common etiologies in Romanian intrahospital infections, finding efficient alternatives in fighting such infections is a national and international priority. 

Our study has shown that two essential oils, namely, tea tree and thyme, are particularly effective against *P. aeruginosa* strains, regardless of their isolation source and antibiotic resistance. These EOs showed much-reduced MIC values and were able to inhibit biofilm formation when utilized in sub-inhibitory concentrations. 

Although preliminary, our results reveal that plant-derived EOs could be efficiently utilized for the therapy of *P. aeruginosa* infections, covering susceptible and resistant strains, as well as planktonic- or biofilm-embedded bacteria. This study supports the idea that natural plant compounds and EOs are a resourceful choice for the development of efficient antimicrobials, which could be intensively exploited by future studies to develop efficient and ecologic antimicrobials, based on natural products, such as plant extracts and EOs.

## Figures and Tables

**Table 1:** *P. aeruginosa* strain codes and isolation sources.

Strain code	Source	Strain code	Source
Ps. 1	ATCC 27853, US	Ps. 13	urban wastewater
Ps. 2	PAO1, ATCC, US	Ps. 14	urban wastewater
Ps. 3	intrahospital infection	Ps. 15	urban wastewater
Ps. 4	intrahospital infection	Ps. 16	urban wastewater
Ps. 5	intrahospital infection	Ps. 17	urban wastewater
Ps. 6	intrahospital infection	Ps. 18	urban wastewater
Ps. 7	intrahospital infection	Ps. 19	urban wastewater
Ps. 8	intrahospital infection	Ps. 20	urban wastewater
Ps. 12	intrahospital infection	Ps. 25	urban wastewater
Ps. 9	hospital sewage	Ps. 26	urban wastewater
Ps. 10	hospital sewage	Ps. 27	urban wastewater
Ps. 11	hospital sewage	Ps. 28	urban wastewater
Ps. 21	hospital sewage	Ps. 29	urban wastewater
Ps. 22	hospital sewage	Ps. 30	urban wastewater
Ps. 23	hospital sewage	Ps. 31	urban wastewater
Ps. 24	hospital sewage	Ps. 32	urban wastewater
Ps. 33	hospital sewage	Ps. 34	urban wastewater
Ps. 36	hospital sewage	Ps. 35	urban wastewater

**Table 2:** Primers for virulence gene detection.

Virulence Genes	Primers	Primer Sequences	Amplicon Size (bp)
*plcH*	plcH-F plcH-R	5′ GAAGCCATGGGCTACTTCAA 3′ 5′ AGAGTGACGAGGGGTAG 3′	466
*plcN*	plcN-F plcH-R	5′ GTTATCGCAACCAGCCCTAC 3′ 5′ AGGTCGAACACCTGGAACAC 3′	307
*exoU*	exoU-F exoU-R	5′ CCGTTGTGGTGCCGTTGAAG 3′ 5′ CCAGATGTTCACCGACTCG 3′	134
*exoT*	exoT-F exoT-R	5′ AATCGCCGTCCAACTGCATGCG 3′ 5′ TGTTCGCCGAGGTACTGCTC 3′	152
*exoS*	exoS-F exoS-R	5′ ATCGCTTCAGCAGAGTCCGTC 3′ 5′ CAGGCCAGATCAAGGCCGCGC 3′	1352
*TCF/TCR*	TCF/R-F TCF/R-R	5′ TATTTCGCCGACTCCCTGTA 3′ 5′ GAATAGACGCCGCTGAAATC 3′	752
*algD*	algD-F algD-R	5′ ATGCGAATCAGCATCTTTGGT 3′ 5′ CTACCAGCAGATGCCCTCGGC 3′	1310

**Table 3:** Amplification program for virulence genes.

Genes	Amplification Program					
	Denaturation	No. of Cycles	Denaturation	Annealing	Extension	Final Extension
*plcH*	94 °C, 3 min	30	94 °C, 30 s	55 °C, 1 min	72 °C, 1.5 min	72 °C, 5 min
*plcN*	94 °C, 3 min	30	94 °C, 30 s	55 °C, 1 min	72 °C, 1.5 min	72 °C, 5 min
*exoU*	94 °C, 2 min	30	94 °C, 30 s	59 °C, 30 s	68 °C, 1 min	68 °C, 7 min
*exoT*	94 °C, 2 min	30	94 °C, 30 s	59 °C, 30 s	68 °C, 1 min	68 °C, 7 min
*exoS*	94 °C, 5 min	35	94 °C, 30 s	65 °C, 30 s	72 °C, 2 min	72 °C, 7 min
*TCF/TCR*	94 °C, 5 min	30	94 °C, 30 s	60 °C, 30 s	72 °C, 2 min	72 °C, 5 min
*algD*	90 °C, 3 min	30	94 °C, 30 s	51 °C, 1 min	72 °C, 1.5 min	72 °C, 5 min

**Table 4:** Primers for the detection of antibiotic resistance genes.

ARGs	Primers	Primer Sequences	Amplicon Size (bp)
*bla*_VIM_	IMP-F IMP-R	5′ GATGGTGTTTGGTCGCATA 3′ 5′ CGAATGCGCAGCACCAG 3′	390
*bla*_IMP_	IMP-F IMP-R	5′ GGAATAGAGTGGCTTAAYTCTC 3′ 5′ GGTTTAAYAAAACAACCACC 3′	232
*bla*_TEM_	TEM-F TEM-R	5′ ATAAAATTCTTGAAGACGAAA 3′ 5′ GTCAGTTACCAATGCTTAATC 3′	1080
*bla*_CTX-M_	CTX-M-F CTX-M-R	5′ CGCTGTTGTTAGGAAGTGTG 3′ 5′ GGCTGGGTGAAGTAAGTGAC 3′	754
*bla*_SHV_	SHV-F SHV-R	5′ TGGTTATGCGTTATATTCGCC 3′ 5′ GGTTAGCGTTGCCAGTGCT 3′	868
*bla*_VEB_	VEB-F VEB-R	5′ CGACTTCCATTTCCCGATGC 3′ 5′ GGACTCTGCAACAAATACGC 3′	390
*bla*_GES_	GES-C GES-D	5′ GTT TTG CAA TGT GCT CAA CG 3′ 5′ TCG CAT AGC AAT AGG CGT AG 3′	371
*bla*_PER_	PER-F PER-R	5′ AATTTGGGCTTAGGGCAGAA 3′ 5′ ATGAATGTCATTATAAAAGC 3′	925
*bla*_NDM_	NDM-F NDM-R	5′ GGTTTGGCGATCTGGTTTTC 3′ 5′ CGGAATGGCTCATCACGATC 3′	621
*aac-3-Ia*	aac-3-Ia F aac-3-IaR	5′-ATGGGCATCATTCGCACA-3′ 5′-TCTCGGCTTGAACGAATTGT-3′	484
*qnrA*	qnrA-F qnrA-R	5′ AGAGGATTTCTCACGCCAGG 3′ 5′ TGCCAGGCACAGATCTTGAC	580
*qnrB*	qnrB-F qnrB-R	5′ GGMATHGAAATTCGCCACTG 3′ 5′ TTTGCYGYYCGCCAGTCGAA	264
*qnrS*	qnrS-F qnrS-R	5′ GCAAGTTCATTGAACAGGGT 3′ 5′ TCTAAACCGTCGAGTTCGGCG 3′	428

**Table 5:** Amplification program for antibiotic resistance genes.

Gene	Amplification Program					
	Denaturation	No. of Cycles	Denaturation	Annealing	Extension	Final Extension
*bla*_VIM_	94 °C, 10 min	36	95 °C, 30 s	55 °C, 40 s	72 °C, 5 min	72 °C, 10 min
*bla_I_*_MP_	94 °C, 10 min	36	95 °C, 30 s	55 °C, 40 s	72 °C, 5 min	
*bla*_TEM_	95 °C, 5 min	30	95 °C, 30 s	52 °C, 40 s	72 °C, 70 s	
*bla*_CTX-M_	95 °C, 5 min	30	95 °C, 30 s	56 °C, 40 s	72 °C, 60 s	
*bla*_SHV_	95 °C, 5 min	30	95 °C, 30 s	56 °C, 40 s	72 °C, 60 s	
*bla*_VEB_	95 °C, 5 min	30	95 °C, 30 s	56 °C, 40 s	72 °C, 50 s	
*bla*_GES_	95 °C, 5 min	30	95 °C, 30 s	56 °C, 40 s	72 °C, 50 s	
*bla*_PER_	95 °C, 5 min	30	95 °C, 30 s	50 °C, 40 s	72 °C, 50 s	
*bla*_NDM_	95 °C, 5 min	30	95 °C, 30 s	55 °C, 40 s	72 °C, 50 s	
*aac-3-Ia*	94 °C, 5 min	35	94 °C, 30 s	55 °C, 30 s	72 °C, 1 min	
*qnrA*	95 °C, 10 min	30	95 °C, 30 s	52 °C, 40 s	72 °C, 5 min	
*qnrB*	95 °C, 10 min	30	95 °C, 1 min	54 °C, 1 min	72 °C, 10 min	
*qnrS*	94 °C, 10 min	30	95 °C, 30 s	52 °C, 40 s	72 °C, 5 min	
